# Highly efficient single-junction GaAs thin-film solar cell on flexible substrate

**DOI:** 10.1038/srep30107

**Published:** 2016-07-20

**Authors:** Sunghyun Moon, Kangho Kim, Youngjo Kim, Junseok Heo, Jaejin Lee

**Affiliations:** 1 Department of Electrical and Computer Engineering, Ajou University, Suwon 443-749, Republic of Korea

## Abstract

There has been much interest in developing a thin-film solar cell because it is lightweight and flexible. The GaAs thin-film solar cell is a top contender in the thin-film solar cell market in that it has a high power conversion efficiency (PCE) compared to that of other thin-film solar cells. There are two common structures for the GaAs solar cell: n (emitter)-on-p (base) and p-on-n. The former performs better due to its high collection efficiency because the electron diffusion length of the p-type base region is much longer than the hole diffusion length of the n-type base region. However, it has been limited to fabricate highly efficient n-on-p single-junction GaAs thin film solar cell on a flexible substrate due to technical obstacles. We investigated a simple and fast epitaxial lift-off (ELO) method that uses a stress originating from a Cr/Au bilayer on a 125-μm-thick flexible substrate. A metal combination of AuBe/Pt/Au is employed as a new p-type ohmic contact with which an n-on-p single-junction GaAs thin-film solar cell on flexible substrate was successfully fabricated. The PCE of the fabricated single-junction GaAs thin-film solar cells reached 22.08% under air mass 1.5 global illumination.

In the last few decades, much effort has gone into developing a thin-film solar cell to take advantage of its light weight and flexibility. A variety of light-absorbing materials such as amorphous Si (a-Si), compound semiconductors, and organic materials have been investigated for use in thin-film solar cells. In particular, a GaAs-based thin-film solar cell could be the leader of the future thin-film solar cell market because of its unri**v**aled high efficiency (28.8%, Alta Devices, Sunnyvale, CA, USA^1^), long-term stability, and reasonable cost^2^. Konagai *et al*.^3^ developed the Peeled Film Technology (PFT) as the epitaxial lift-off (ELO) method for fabricating GaAs thin-film solar cells firstly. In the ELO method, the thin film is separated from the substrate by selectively etching a sacrificial layer between the active region and the substrate. This allows the transfer of the thin film to a foreign carrier and makes detached bulk substrate reusable. Manufacturing costs are significantly reduced by reusing the GaAs substrate^4^,^5^,^6^. Because of these advantages, the ELO method for fabricating GaAs thin-film solar cells has been widely investigated. Wu *et al*. fabricated a GaAs thin-film solar cell with an electroplated Cu substrate via a cross-shape-patterned ELO method that included hydrofluoric (HF) acid solutions mixed with hydrophilic solvent^7^. However, a thin-film solar cell with an electroplated Cu substrate is not very flexible and has a low power-to-weight ratio^8^ due to its considerable weight, thus diminishing the advantages of the thin-film solar cell. Tatavarti *et al*. reported on an n-on-p single-junction GaAs thin-film solar cell grown by metal organic chemical vapor deposition (MOCVD)^9^. The fabricated thin film solar cell with a thick metal handler showed a power conversion efficiency (PCE) of 21.11%, but its weight is relatively heavy. Recently, Lee *et al*. described an ELO method that utilizes a tensile stress resulting from the deposition of Ir/Au on a 50-μm-thick Kapton sheet, and a cold-welding technique without adhesive^10^. Because of a lack of an HF-resistant p-contact metal this simple ELO method could be applied only to the p (emitter)-on-n (base) solar cell structure, where the Be-doped p-GaAs layer has to be grown prior to the n-GaAs layer using molecular beam epitaxy (MBE). Hence, a high diffusion of Be from the p-GaAs layer is inevitable while growing the n-GaAs layer at an elevated temperature^11^,^12^ and a resultant PCE was relatively low, 18.1%.

In the present paper, we have demonstrated a highly efficient GaAs thin-film solar cell fabricated on a flexible substrate using a simple and fast ELO method that utilizes the stress of a sputtered Cr/Au bilayer. In order to realize the highly efficient and flexible thin film solar cell, an HF-resistant AuBe/Pt/Au metal combination is employed as a new p-type ohmic contact, by which an n (emitter)-on-p (base) single-junction GaAs thin-film solar cell has been successfully fabricated. The inverted structures of GaAs solar cell grown using MOCVD becomes a conventional n-on-p structure after ELO process. The single-junction GaAs thin-film solar cell on a flexible substrate is made using the standard solar cell fabrication process.

## Results

**Figure 1:**
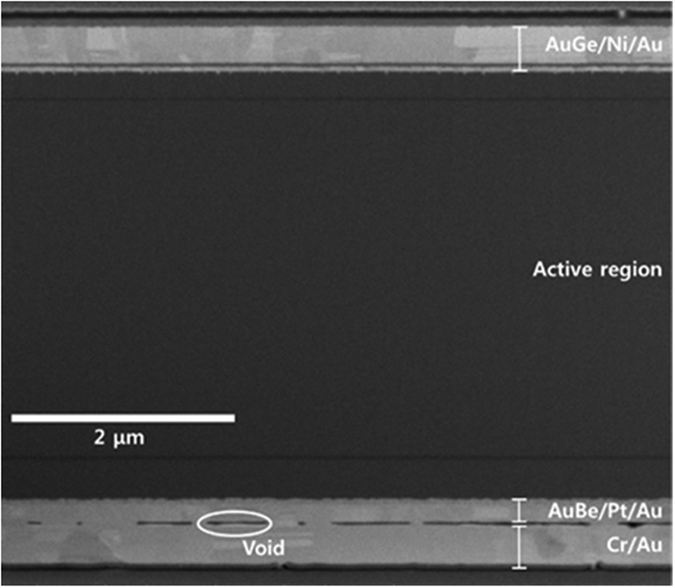
Both n- and p-ohmic metal layers as well as the GaAs active region are clearly visible in this cross-sectional SEM image of a fabricated GaAs thin-film solar cell obtained using a focused ion beam (FIB).

Figure 1 shows a cross-sectional scanning electron microscopy (SEM) image, obtained using focused ion beam (FIB) milling, of the fabricated GaAs thin-film solar cell on a flexible substrate. Both n- and p-ohmic metal layers are clearly seen as well as the GaAs active region. Although a few voids are observed at the metal bonding interface, they probably do not contribute to an increase in series resistance because most of the areas are in contact with a highly conducting metal layer.

**Figure 2:**
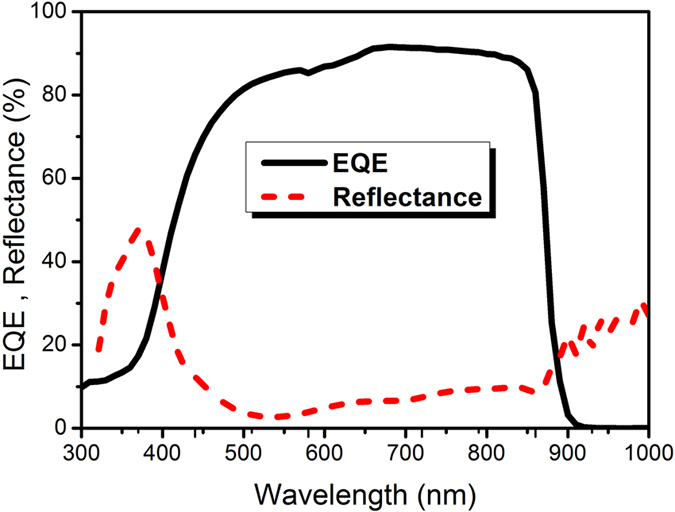
EQE and reflectance of the GaAs thin-film solar cell.

Figure 2 shows the measured external quantum efficiency (EQE) and reflectance curves of the fabricated GaAs thin-film solar cell. The sudden increase in the spectral response of the EQE around 880 nm corresponds to the band gap energy of GaAs. The high EQE of >80% occurs in the wavelength range of 500–800 nm. The cutoff seen below about 470 nm is generally explained by the inefficient extraction of carriers generated near the window and emitter layers. In addition, the broad reflection peak seen in the figure is also responsible to make the EQE drop more rapidly. The delicate optimization of the antireflection coatings (ARCs) potentially improves the efficiency of the solar cell further.

**Figure 3:**
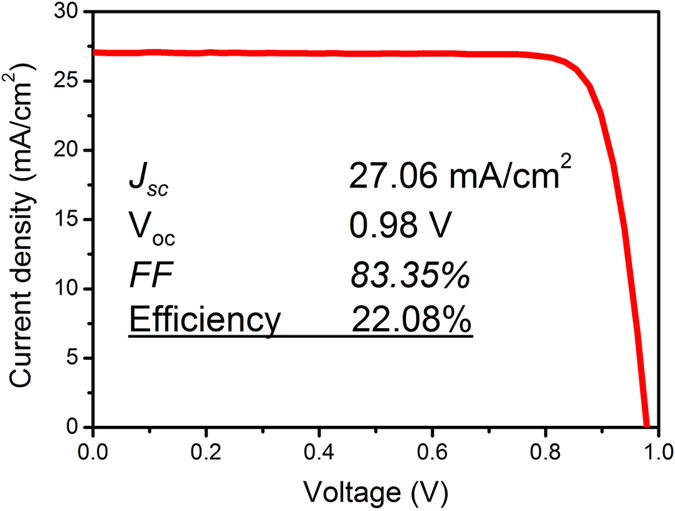
*J−V* curve of the GaAs thin-film solar cell with a conversion efficiency of 22.08%. The fabricated device has a *J*_sc_ of 27.06 mA/cm^2^, a *V*_oc_ of 0.98 V, and a *FF* of 83.35%.

Figure 3 shows the current density−voltage (*J*−*V*) characteristics of a complete single-junction GaAs thin-film solar cell measured at room temperature (25 °C) and AM 1.5 G illumination. The high *FF* is attributed to a low contact resistivity of p-ohmic contact. The contact resistivity measured by a transmission line model (TLM) method^13^ is 4 × 10^−4^ Ω·cm^2^. It is worth mentioning that the estimated PCE of 22.08% is much higher than that of the p-on-n solar cell fabricated on a flexible substrate^10^.

## Discussion

Table 1 summarizes the characteristics and structures of GaAs thin-film solar cells reported in published studies and this work. In general, a single-junction solar cell consists of a highly doped emitter layer and a lightly doped base layer. Hence, a long minority-carrier diffusion length in the base region is an extremely important factor that determines the collection efficiency of photogenerated carriers. The minority-carrier diffusion length *L*_D_ depends on the lifetime and mobility of the minority carrier and is expressed by 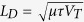, where *μ* is the carrier mobility, *τ* is the minority-carrier lifetime, and *V*_T_ is the thermal voltage. Therefore, p-GaAs is preferred for the base region because electrons have greater mobility than holes in GaAs. Thus, the n-on-p structure has better collection efficiency in the GaAs p-n junction than the p-on-n structure^14^,^15^. Despite the advantages of the n-on-p structure, its implementation on a flexible substrate using the ELO method has been limited because of the absence of an HF-resistant p-ohmic contact with low contact resistance. It is worth noting that the work done by Lee *et al*.^10^ showed a relatively low efficiency and a low *FF* that are not only due to more growth defects in the MBE-grown layer^16^ but mostly due to an inherent low-collection efficiency in the p-on-n structure. Tatavarti *et al*. grew an n-on-p structure by MOCVD and then transferred the active layer onto the thick metal handle carrier^9^. The fabricated cell was relatively heavy and showed a lower PCE of 21.11%. The thin film solar cell fabricated in Alta devices has a high PCE^17^ and a remarkable *V*_oc_ of 1.12 V probably due to photon recycling as well as the optimized design^18^, but its detailed structure has not been reported. In this work, we presented a new combination of AuBe/Pt/Au as the HF-resistant p-ohmic contact, where Pt acts as the barrier to impede Au diffusion during the bonding process^19^. In result, the thin film solar cell with the n-on-p structure was successfully fabricated on flexible substrate which exhibits a PCE of 22.08%. We have measured all the GaAs cells properly fabricated on 2 inch wafer. The efficiency was slightly varied from cell to cell over the whole wafer but the most of cells exhibit efficiencies of more than 21% and the minimum efficiency was at least 20.84%. Thus, these highly efficient GaAs solar cells are reliably demonstrated.

**Figure 4:**
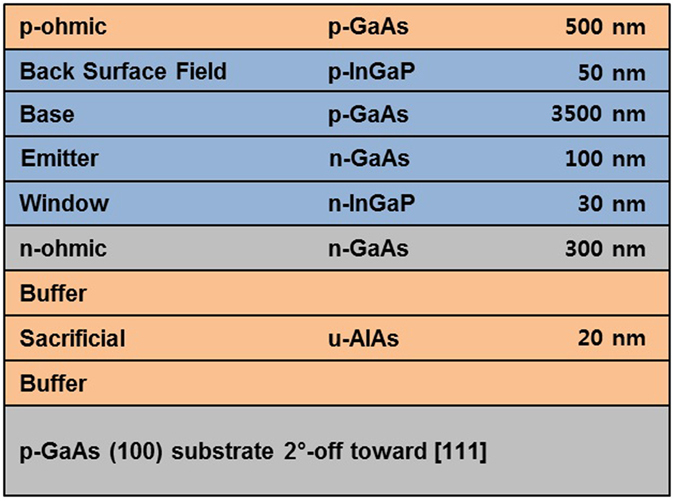
Heterostructure of inverted single-junction GaAs thin-film solar cell.

The heterostructure of the GaAs solar cell shown in Fig. 4 was not fully optimized as a thin-film solar cell to maximize the efficiency. Unlike with a bulk-type solar cell, optimizing photon management can enhance the collection efficiency of a thin-film solar cell^20^,^21^. A short base with a highly reflective back contact enables a long-wavelength photon to be absorbed near the p-n junction by reflection at the back contact. As a result, *V*_oc_ and *J*_sc_ improve in accordance with the increase in collection efficiency, thus showing that the fabricated GaAs thin-film solar cell is a high-performance device. The GaAs thin-film solar cell presented here has a relatively thick base compared to that of the other cells, as found in Table 1. The long-wavelength photons might be absorbed further from the depletion region, upon which the collection efficiency exponentially decreases^21^. Therefore, it could have a higher conversion efficiency by reducing the thickness of the base region and enhancing the reflectivity of the p-ohmic contact.

## Conclusions

We have successfully demonstrated the fabrication of a highly efficient n-on-p single-junction GaAs thin-film solar cell on a flexible substrate using the simple ELO method. The n-on-p structure is preferred over the p-on-n structure because of the higher charge collection efficiency of electrons with a long diffusion length in the p-base region. However, the typical p-ohmic contact is vulnerable to the HF-based etchant, which limits the implementation of the n-on-p structure on a flexible substrate. To address this problem, we used the combination of AuBe/Pt/Au as a new p-ohmic metal that can withstand the ELO process and has a contact resistivity as low as 4 × 10^−4^ Ω·cm^2^. The characteristics of the fabricated GaAs thin-film solar cell on flexible substrates were investigated by measuring the reflectance, EQE, and photovoltaic *J*−*V* curves. Its performance characteristics were, *J*_sc_ = 27.06 mA/cm^2^, *V*_oc_ = 0.98 V, *FF* = 83.35%, and PCE = 22.08%. The efficiency can be increased by optimizing the base region and the metal reflector on the backside of the thin film. The optimized, high-efficiency thin-film GaAs solar cells fabricated using this mass-production-friendly technology will enable the widespread use of III-V thin-film solar cells in industrial and commercial applications.

## Methods

**Figure 5:**
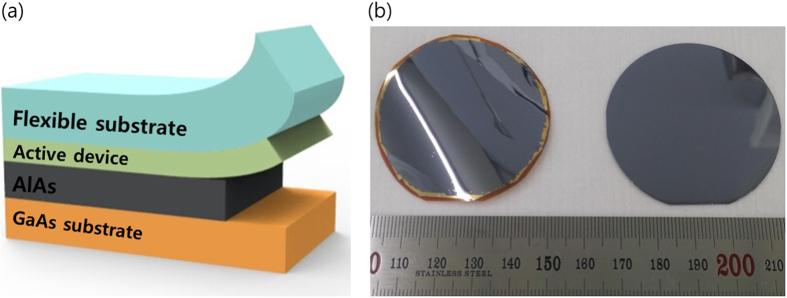
(**a**) Schematic diagram of the simple and fast ELO method. (**b**) Photographs of the epilayer on the flexible substrate and growth-ready substrate that can be reused after the ELO process. The GaAs solar cell epilayer bonded on the flexible substrate is separated from the substrate by etching an AlAs sacrificial layer in a mixture of hydrophilic and HF acid. The Cr/Au layer deposited on the polymer substrate makes it easier to separate the epilayer from the substrate. After the ELO process, the buffer layers are selectively etched by an acid solution, thereby revealing the shiny and smooth surface of both the thin film and the GaAs substrate.

**Figure 6:**
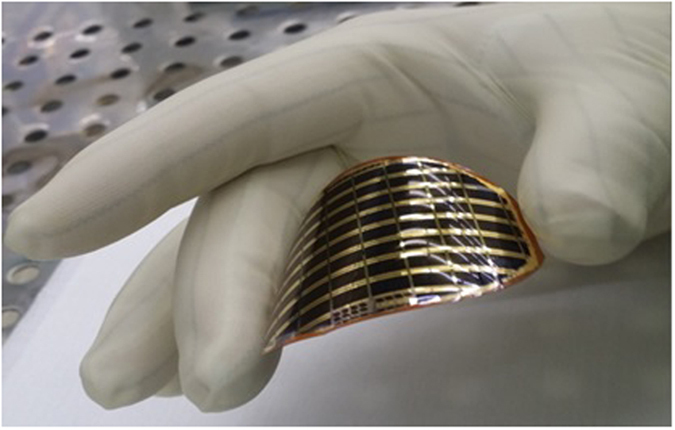
Photograph of fabricated single-junction GaAs thin-film solar cell on flexible substrates.

The structures of the thin-film solar cell are grown via MOCVD on a Zn-doped, p-type GaAs (100) substrate with a misorientation of 2° off toward [111] direction. The heterostructure of the inverted single-junction GaAs solar cell is shown in Fig. 4. The buffer layers are grown on both the top and bottom of the 20-nm-thick AlAs, by doing so, both of the surface of the epilayer and the substrate are protected from byproducts generated after and during the ELO process^6^,^22^,^23^. For the active region, the n-GaAs layer is grown prior to the p-GaAs layer, resulting in an n-on-p structure after the ELO process. Hence, an HF-resistant p-ohmic contact must be deposited on top of the as-grown heterostructure. The p-ohmic contact widely used in GaAs solar cells includes Ti^24^,^25^,^26^,^27^, which is easily etched in an HF acid. To address the vulnerability of the p-ohmic contact, we use the AuBe/Pt/Au metal combination that remains intact in the HF solution during the ELO. After deposition of a Cr/Au bilayer on the 125-μm-thick flexible substrate, the metal-to-metal bonding is processed by controlling the temperature and pressure. The GaAs solar cell epilayer, bonded onto the flexible substrate, is separated from the substrate by etching an AlAs sacrificial layer in an HF-based etchant, as shown in Fig. 5(a). The Cr/Au bilayer-induced stress makes the transfer of the epilayer to the flexible substrate easier. The HF acid etchant mixed with hydrophilic substance increases the etch rate of the AlAs layer because the chemical reaction generates less residue, which helps the inward diffusion of the etchant^7^. After the ELO process, the buffer layers which are exposed on both the thin film and the detached substrate are selectively etched by a phosphoric acid, thereby preparing a fresh GaAs surface on which to deposit front n-metal structures and the substrate for reuse. Figure 5(b) shows the thin film transferred onto a flexible substrate and the growth-ready recycled substrate. A grid pattern of n-ohmic contact, consisting of AuGe/Ni/Au, is deposited on the thin film by e-beam evaporation. Then a citric acid is used to selectively remove the n-GaAs layer to expose an InGaP window layer, followed by mesa etching to isolate the cell. Finally, ARCs of MgF_2_/ZnS are deposited on the InGaP layer^28^. The fabricated cell size is 0.20 cm^2^. Figure 6 is a photograph of the fabricated GaAs thin-film solar cell, the performance of which was measured using a class A solar simulator with a xenon lamp under air mass 1.5 global (AM 1.5 G) illumination. The irradiance of the simulator was calibrated using a reference cell to verify the AM 1.5 G illumination before measuring the fabricated solar cell.

## Additional Information

**How to cite this article**: Moon, S. *et al*. Highly efficient single-junction GaAs thin-film solar cell on flexible substrate. *Sci. Rep.* **6**, 30107; doi: 10.1038/srep30107 (2016).

## Supplementary Material

Supplementary Information

## Figures and Tables

**Table 1:** Summary of characteristics and structures of GaAs thin-film solar cells from published reports and this work.

Growth system	*J*_*sc*_(mA/cm^2^)	*V*_*oc*_(V)	*FF*(%)	Efficiency (%)	Structure	Base thickness (μm)	Bonding method/Carrier	Authors (year )
MOCVD	24.57	1.01	85.25	21.11	n-on-p	2	Deposition/Metal	Tatavarti *et al*.^9^
MOCVD	29.67	1.12	86.50	28.80	Unknown	Unknown	Adhesive bonding/Polymer	Alta devices Inc.^17^(2012)
MOCVD	19.61	1.00	81.48	15.98	n-on-p	1.5	Electroplating/Metal	Wu *et al*.^7^(2013)
MBE	24.2	0.98	76.4	18.1	p-on-n	3	Metal bonding/Polymer	Lee *et al*.^10^(2014)
MOCVD	27.06	0.98	83.35	22.08	n-on-p	3.5	Metal bonding/Polymer	Moon *et al*. (this work) (2016)
