# Supplementary material for: Highly efficient single-junction GaAs thin-film solar cell on flexible substrate
Source: Sci Rep. 2016 Jul 20;6:30107. doi: 10.1038/srep30107 (PMC4951801; doi:10.1038/srep30107)
Supplement: Supplementary Information [file srep30107-s1.pdf]

Highly efficient single-junction GaAs thin-film solar cell on flexible substrate

Sunghyun Moon, Kangho Kim, Youngjo Kim, Junseok Heo* & Jaejin Lee*

Department of Electrical and Computer Engineering, Ajou University,

Suwon 443-749, Republic of Korea

1. Characterization of stress in as-deposited Cr layer

We investigated a simple and fast epitaxial lift-off (ELO) method that uses a stress originating from a Cr/Au (500/3000 Å) bilayer on a 125-μm-thick flexible substrate. The Cr/Au bilayer is prepared by reactive DC-sputtering magnetron equipment (SME-200J), and its induced-stress makes the transfer of epilayer to a flexible substrate easier owing to an inward diffusion of the etchant during ELO process. It is worth noting that a moderate metal stress is helpful to transfer the epilayer to a flexible substrate fast and intactly. In general, the stress of sputtered thin film can be controlled upon change of sputtering conditions such as power and pressure[1].

In order to investigate the stress of Cr/Au bilayer, a 500 Å-thick Cr layer was sputtered on Si substrate with different powers while keeping an Ar gas pressure constant. The stress of as-deposited metal was characterized by using a stress measurement system (FSM 500TC). Figure 1 shows a diagram of the FSM 500TC equipped with Non-Destructive Optilever™ Laser Scanning technique to measure the change of curvature in a wafer deformed by a sputtered Cr layer atop. The laser scanning on the surface of the wafer gives a precise position at high speed. A bare Si wafer is measured prior to Cr deposition as a reference. The radius of curvature (R) is determined by comparing the position data measured on the Cr-deposited sample with the reference. Then, the film stress, S is calculated by $S = \frac{ED^2}{6(1-V)RT}$ where E = Young's modulus of the substrate, V = Poisson's ratio of the substrate, D = Thickness of the substrate, and T = Thickness of the film ($T \ll D$)[2].

Table 1 shows the strain characterization of the sputtered Cr layer on Si substrates. The measured stress of as-deposited samples is identified as compressive strain. It is also observed that strain increases as a sputtering power increases. The demonstrated n-on-p single-junction GaAs thin-film solar cell employs a sputtering power of 500 W to utilize a moderate compressive strain for an efficient epitaxial lift-off (ELO) process.

Figure 1. Diagram of the stress measurement tool (FSM 500TC)

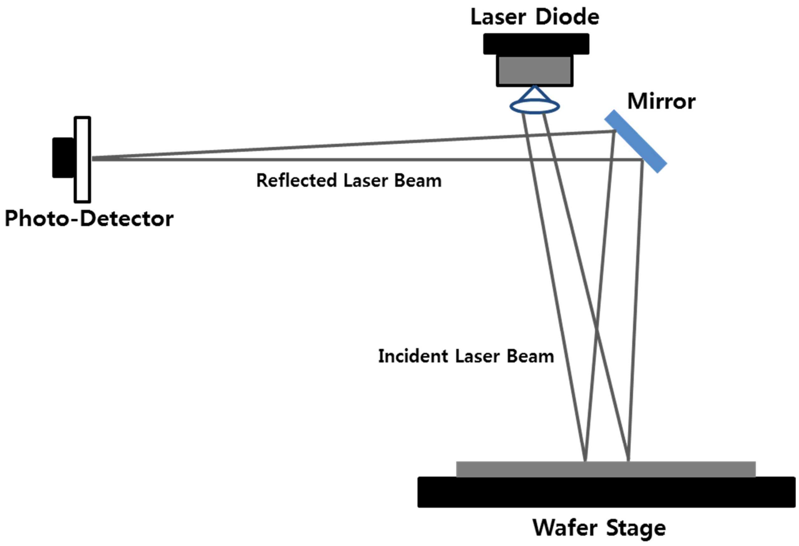

Table 1. Metal stress of as-sputtered 500 Å-thick Cr with sputtering powers of 500 and 1000 W

Sputtering power (W)	Substrate	Stress (MPa)	Radius (m)	Temp (°C)	Film thickness (Å)	Wafer thickness (mm)
500	Si (bare)	0	705.88	23	0	0.55
	Si (Cr-deposited)	1585	96.81	24	500	0.55
1000	Si (bare)	0	20095.44	23	0	0.55
	Si (Cr-deposited)	1940	91.58	24	500	0.55

2. Characterization of p-ohmic contact resistance

The absence of an HF-resistant p-ohmic contact with low contact resistance is a major obstacle to fabricate a highly efficient n-on-p GaAs thin-film solar cell. In this study, the HF-resistant metal combination of AuBe/Pt/Au was suggested and characterized by a contact resistivity as low as $4 \times 10^{-4}\ \Omega\cdot cm^2$. A contact resistivity is determined by a circular transfer length method (CTLM) measurement technique[3] as shown in Figure 2. The outer circular contact has a constant radius of 100 μm, while the different gaps between inner and outer circle contacts are 5, 10, 15, 20, 30 and 40 μm. The 6 separated measurements using a probe station (Summit11741B) are performed. The measured CTLM data can be transformed into linear relationship via correction factors from which more accurate contact resistivity is extracted[4]. Table 2 shows the correction factors for 6 different interval spacing (5−40 μm). A slight difference between experimental data and compensated values for linear fit can be checked as shown in Figure 3.

Figure 2. The image of CTLM with various gap spacing between inner and outer circle contacts

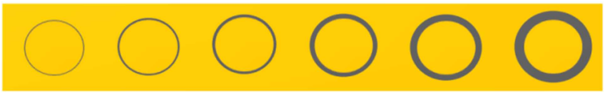

Figure 3. A graph of total resistance versus gap spacing before (circle) and after (square) treatment with correction factors

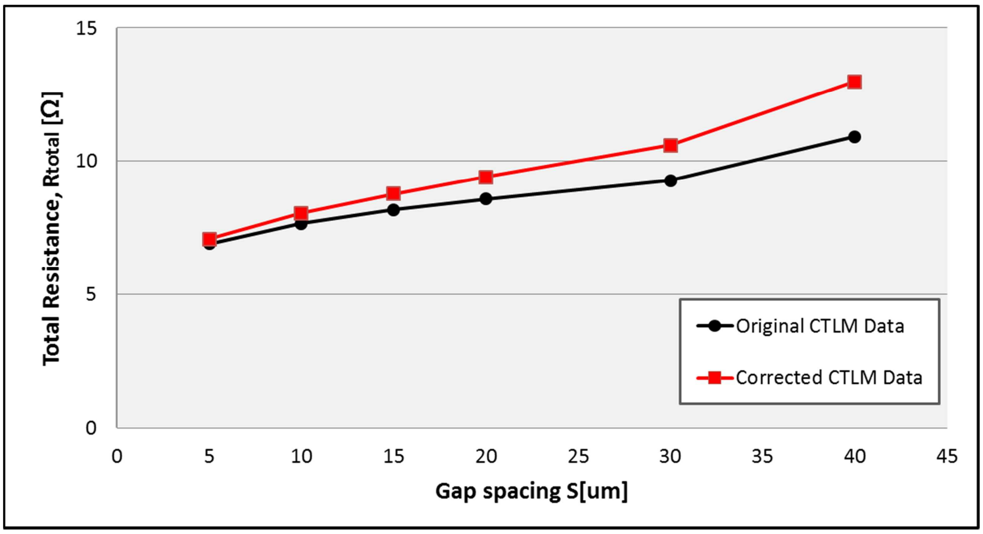

Table 2. Correction factors for 6 different interval spacing

Gap spacing (μm)	Correction factors	Gap spacing (μm)	Correction factors
5	0.976	20	0.911
10	0.953	30	0.875
15	0.931	40	0.841

3. References

[1] Pethe, S. A., Takahashi, E., Kaul A. & Dhere N. G. Effect of sputtering process parameters on film properties of molybdenum back contact. *Sol. Energ. Mat. Sol. Cells*. **100**, 1-5 (2012).

[2] FSM500 Technical Manual, Frontier Semiconductor Inc., San Jose, CA, USA

[3] Jacobs, B., Kramer, M.C.J.C.M., Geluk, E.J. & Karouta, F. Optimisation of the Ti/Al/Ni/Au ohmic contact on AlGaN/GaN FET structures. *J. Cryst. Growth*. **241**, 15-18 (2002).

[4] Klootwijk, J.H. & Timmering, C.E. Merits and limitations of Circular TLM structures for contact resistance determination for novel Ⅲ-Ⅴ HBTs, Paper presented at *IEEE Int. Conf.* Microelectronics Test Structures (ICMTS) (10.1109/ICMTS.2004.1309489) (2004, March).
